# Temperature dependence of erythromelalgia mutation L858F in sodium channel Nav1.7

**DOI:** 10.1186/1744-8069-3-3

**Published:** 2007-01-19

**Authors:** Chongyang Han, Angelika Lampert, Anthony M Rush, Sulayman D Dib-Hajj, Xiaoliang Wang, Yong Yang, Stephen G Waxman

**Affiliations:** 1 Institute of Materia Medica, Chinese Academy of Medical Sciences and Peking Union Medical College, Beijing, 100050, China; 2 Department of Neurology; 3 Center for Neuroscience and Regeneration Research, Yale University School of Medicine, New Haven, CT 06510, USA; 4 Rehabilitation Research Center, Veterans Affairs Connecticut Healthcare Center, West Haven, CT 06516, USA; 5 Department of Dermatology, Peking University First Hospital, Beijing, China, 100034; 6 NeuroSolutions Ltd., PO Box 3517, Coventry CV4 7ZS, UK

## Abstract

**Background:**

The disabling chronic pain syndrome erythromelalgia (also termed erythermalgia) is characterized by attacks of burning pain in the extremities induced by warmth. Pharmacological treatment is often ineffective, but the pain can be alleviated by cooling of the limbs. Inherited erythromelalgia has recently been linked to mutations in the gene SCN9A, which encodes the voltage-gated sodium channel Nav1.7. Nav1.7 is preferentially expressed in most nociceptive DRG neurons and in sympathetic ganglion neurons. It has recently been shown that several disease-causing erythromelalgia mutations alter channel-gating behavior in a manner that increases DRG neuron excitability.

**Results:**

Here we tested the effects of temperature on gating properties of wild type Nav1.7 and mutant L858F channels. Whole-cell voltage-clamp measurements on wild type or L858F channels expressed in HEK293 cells revealed that cooling decreases current density, slows deactivation and increases ramp currents for both mutant and wild type channels. However, cooling differentially shifts the midpoint of steady-state activation in a depolarizing direction for L858F but not for wild type channels.

**Conclusion:**

The cooling-dependent shift of the activation midpoint of L858F to more positive potentials brings the threshold of activation of the mutant channels closer to that of wild type Nav1.7 at lower temperatures, and is likely to contribute to the alleviation of painful symptoms upon cooling in affected limbs in patients with this erythromelalgia mutation.

## Background

The disabling chronic pain syndrome erythromelalgia (also termed erythermalgia) is characterized by attacks of burning pain in the extremities that are triggered by mild warmth; pharmacological treatment of this disorder is ineffective in many patients [1]. Inherited erythromelalgia (IEM) is transmitted in an autosomal dominant manner [2]. Thus far seven mutations have been reported in SCN9A, the gene which encodes the voltage-gated sodium channel Nav1.7, in familial cases and some sporadic cases (de novo, founder mutations) with IEM [3-8]. Nav1.7 is preferentially expressed in dorsal root ganglion (DRG) and sympathetic ganglion neurons [9-11]. Nav1.7 is present in the majority of nociceptive DRG neurons [12], and has been shown to play an important role in the pathophysiology of inflammatory pain [13,14]. IEM-linked missense mutations in Nav1.7 change gating properties of the channel [4,7,8,15-18] and render DRG neurons hyperexcitable [4,8,18].

Attacks of pain in IEM are alleviated by cooling of the limbs [4-7,19] but the physiological basis for this phenomenon is not understood. Therefore, we investigated the influence of cooling on the biophysical properties of wild type Nav1.7 (WT) and on the Nav1.7 mutation L858F, which has been shown to underlie IEM in Chinese [7] and Canadian [5] families. Using whole-cell patch clamp methods, we have found that cooling differentially affects WT and L858F Nav1.7 channels and diminishes the difference in the voltage-dependence of activation between the two channels, an effect that may contribute to the clinical observation that cooling alleviates pain symptoms of IEM.

## Results

### Current density decreases upon cooling

**Figure 1:**
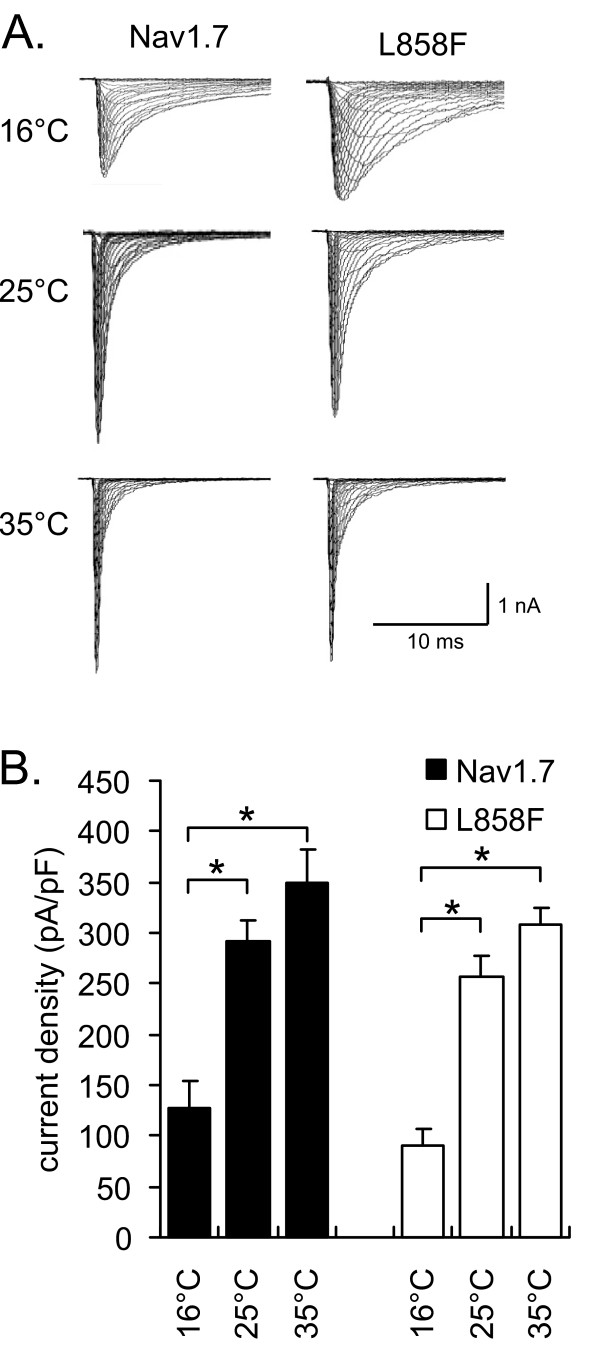
**Cooling decreases current density for Nav1.7 and L858F**. **A. **Representative current-voltage (I-V) families recorded from HEK293 cells stably expressing Nav1.7 (left column) or the mutation L858F (right column) at 16°C, 25°C or 35°C. Cells were held at -120 mV and depolarizing steps were applied to membrane potentials ranging from -80 mV to 40 mV in 5 mV steps. **B**. Temperature dependence of the current density for Nav1.7 (black bars, n = 17, 15, 25) and L858F (white bars, n = 15, 16, 28) at the indicated temperatures. Current density was measured as peak current divided by cell capacitance. * indicate significant differences between values with p < 0.05, tested with ANOVA and Tukey HSD post hoc analysis.

Whole-cell patch-clamp recordings of sodium currents from HEK293 cells stably expressing WT or the IEM mutant Nav1.7 channel (L858F) were carried out at three different temperatures: 16°C, 25°C and 35°C. Both WT and L858F mutant channels produced fast activating and inactivating currents (Figure 1A). The macroscopic opening and closing for WT and L858F channels were both slowed with a reduction in temperature (Figure 1A). While the inactivation time constants were not different between WT and L858F channels, they were significantly slower when the temperature of the recording solution was cooled down for each channel. At a test potential of -25 mV, for example, WT channels inactivated with time constants of 0.35 ± 0.03 ms (35°C, n = 6), 0.92 ± 0.03 ms (25°C, n = 7) and 3.0 ± 0.2 ms (16°C, n = 6), and L858F channels inactivated with time constants of 0.38 ± 0.16 (35°C, n = 8), 1.23 ± 0.03 ms (25°C, n = 10) and 2.5 ± 0.1 ms (16°C, n = 7). Comparison of the peak currents at different temperatures showed a decrease in current density of WT and L858F channels when the temperature was reduced from 35°C or 25°C to 16°C (Figure 1B).

### Steady-state activation shifts with cooling for L858F but not for WT

**Figure 2:**
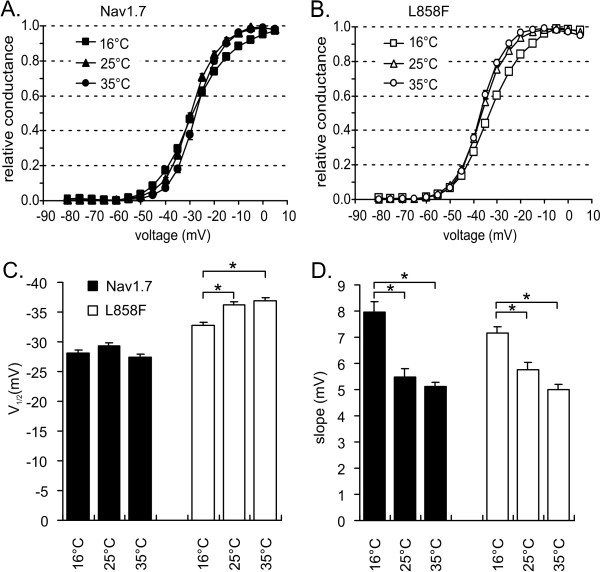
**Activation midpoint shifts to more depolarized potentials upon cooling for L858F, but not for Nav1.7**. **A**. Voltage dependences of conductance for Nav1.7 at 16°C (filled squares, n = 12), 25°C (filled triangles, n = 13) and 35°C (filled circles, n = 12). Conductance curves were derived from current-voltage families, normalized, and fitted with a Boltzmann equation, as described in methods. **B**. Voltage dependences of conductance for L858F at 16°C (open squares, n = 8), 25°C (open triangles, n = 15) and 35°C (open circles, n = 14). **C**. Midpoint of activation for Nav1.7 (black bars) and L858F (white bars) plotted versus temperature. **D**. Slope factor of activation for Nav1.7 (black bars) and L858F (white bars) plotted versus temperature. * indicate significant differences between values with p < 0.05, tested with ANOVA and Tukey HSD post hoc analysis.

We have previously shown that the L858F mutation activates at more negative potentials than Nav1.7 [7]. This is reflected in a negative shift in the midpoint (V_1/2_) of steady-state activation (Figure 2A, B and 2C). We confirmed a significant hyperpolarizing shift in V_1/2 _of activation for L858F compared to WT at all three tested temperatures. WT channels did not show a significant shift in the V_1/2 _of activation at the three temperatures (-27.4 ± 0.6 mV at 35°C, -29.4 ± 0.5 mV at 25°C and -28.1 ± 0.5 mV at 16°C). L858F channels, on the other hand, demonstrated a significant shift of the V_1/2 _of activation to more depolarized potentials when the temperature was lowered to 16°C (-36.9 ± 0.5 mV at 35°C, -36.3 ± 0.5 at 25°C and -32.8 ± 0.5 at 16°C; p < 0.05). Depolarization of the V_1/2 _of activation of L858F brings the activation voltage-dependence of the mutant channel closer to that of WT channels; the difference in V_1/2 _of activation between WT and L858F channels is reduced from 9.6 mV at 35°C, to 4.6 mV at 16°C.

The steepness of the voltage dependence of conductance for WT channels decreases upon cooling, which is reflected in a greater slope factor value (Figure 2D). A similar effect of cooling was seen for L858F. Thus for both WT and L858F, the sensitivity of the channels to small voltage changes close to the midpoint of activation is decreased at low temperatures.

### Steady-state fast inactivation shifts in a similar manner for L858F and WT

**Figure 3:**
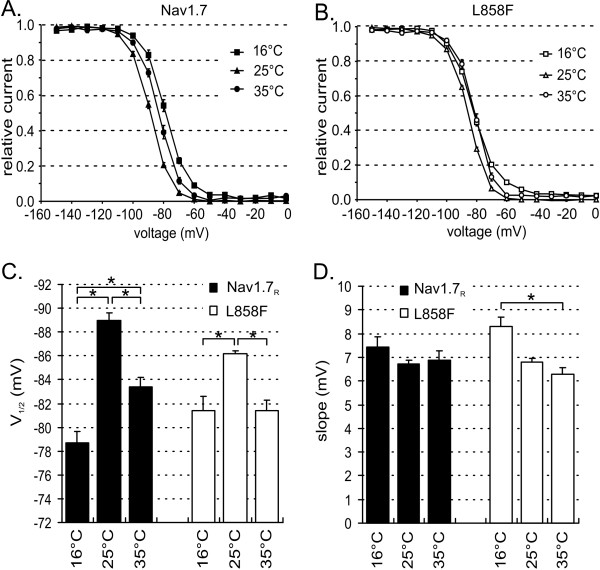
**Steady-state fast inactivation changes in a similar way for Nav1.7 and L858F**. **A**. Voltage dependences of steady-state fast inactivation for Nav1.7 at 16°C (filled squares, n = 12), 25°C (filled triangles, n = 11) and 35°C (filled circles, n = 9). Availability was assessed using a 500 ms prepulse ranging from -150 mV to 0 mV followed by a 40 ms test pulse to -20 mV. The current was normalized to the largest current response evoked by the test pulse. Steady-state inactivation curves were fitted with a Boltzmann equation, as described in methods. **B**. Voltage dependences of steady-state fast inactivation for L858F at 16°C (open squares, n = 9), 25°C (open triangles, n = 11) and 35°C (open circles, n = 10). **C**. Temperature dependence of the midpoint of steady-state inactivation for Nav1.7 (black bars) and L858F (white bars) at the indicated temperatures. **D**. The slope factor of steady-state inactivation for Nav1.7 (black bars) and L858F (white bars) plotted versus temperature. * indicate significant differences between values with p < 0.05, tested with ANOVA and Tukey HSD post hoc analysis.

At 25°C and 35°C L858F showed a significant shift of V_1/2 _of steady-state fast inactivation to more depolarized potentials than WT, comparable to results previously reported [7]. This difference between WT and L858F could not be detected at 16°C (Figure 3A, B and 3C). The V_1/2 _of steady-state fast inactivation is shifted in a hyperpolarized direction by cooling from 35°C to 25°C for both channels {for WT from -84.2 ± 0.7 mV (35°C) to -89 ± 0.6 mV (25°C); for L858F from -81.2 ± 0.6 mV (35°C) to -86.2 ± 0.2 mV (25°C)}.

Interestingly, further cooling of L858F to 16°C induced a depolarizing shift in V_1/2 _of inactivation to a value close to that found at 35°C (-79.5 ± 0.6 mV, 16°C, L858F). The V_1/2 _of steady-state fast inactivation of WT channels at 16°C, however, is shifted to voltages more depolarized than at 35°C (-77.8 ± 0.6 mV, 16°C, WT, Figure 3C). The slope factor of inactivation did not change due to cooling for WT channels, and increases slightly for L858F channels (Figure 3D).

### Ramp currents increase and deactivation slows upon cooling

**Figure 4:**
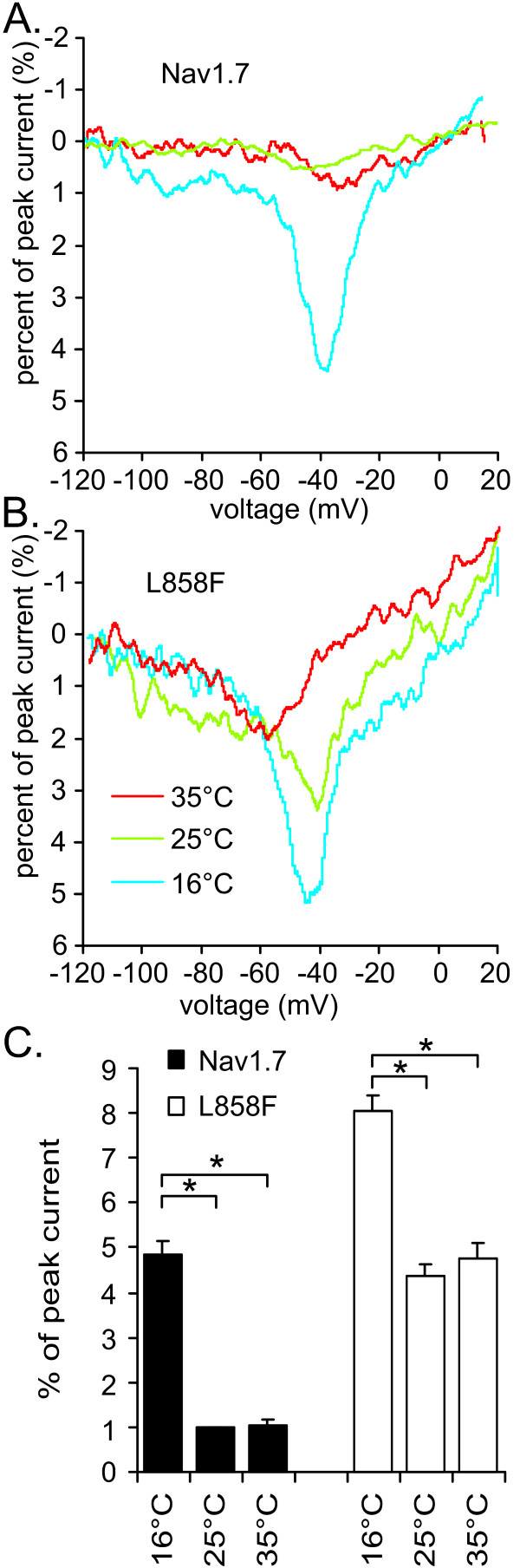
**Cooling increases currents elicited by slow ramp depolarizations, and diminishes the difference between Nav1.7 and L858F**. Representative current traces of Nav1.7 (**A.**) and L858F (**B.**) ramp currents at 16°C, 25°C and 35°C. Cells were held at -120 mV and stimulated with a depolarizing voltage ramp that increased to 20 mV within 600 ms. **C**. The bar graph shows the mean peak currents recorded during the voltage ramps expressed as percent of transient peak current obtained during initial I-V families, 3 min after breaking into the cell; black bars represent Nav1.7 at 16°C (n = 8), 25°C (n = 9) and 35°C (n = 7); white bars represent L858F at 16°C (n = 8), 25°C (n = 9) and 35°C (n = 8). * indicate significant differences between values with p < 0.05, tested with ANOVA and Tukey HSD post hoc analysis.

Application of slow depolarization pulses from a holding potential of -120 mV to +20 mV in 600 ms mimic weak natural stimuli and evoke ramp currents of WT channels as described by Cummins et al. [20] (Figure 4A), and L858F channels as shown by Han et al. [7] (Figure 4B). The ramp current, measured as percentage of transient peak current, increases with cooling for WT channels from 1.05% ± 0.12% (35°C) and 0.98% ± 0.04% (25°C) to 4.87% ± 0.28% (16°C). A similar increase was observed for L858F: 4.75% ± 0.37% (35°C) and 4.36% ± 0.25% (25°C) to 8.07% ± 0.32% (16°C, Figure 4C).

As shown previously [7] L858F produces larger ramp currents than WT channels. The difference in size of the ramp current between WT and L858F channels is significant at each temperature tested. However, the difference between WT and L858F decreases with cooling, which can be seen in the Q_10 _values for WT and L858F. Cooling from 25°C to 16°C shows a Q_10 _value for WT of 5.94, whereas the Q_10 _for L858F is 1.98, indicating a lower temperature sensitivity of the mutant channel compared to WT. Therefore, upon cooling the incremental difference in the size of ramp currents between WT and L858F becomes smaller. Thus, the relative increase in the ramp current for L858F when cooled from 35°C to 16°C (170%) is smaller than the relative increase for WT (463%; Figure 4C).

The increase in ramp current due to cooling could be caused by slowing of deactivation. In order to test the influence of temperature on deactivation kinetics, we measured the deactivation time constants after a brief (0.5 ms) depolarization to -20 mV. At potentials of -40 mV L858F channels deactivate more slowly than WT channels at all three temperatures (deactivation time constant at 16°C: 2.9 ± 0.2 ms WT, 4.7 ± 0.4 ms L858F; at 25°C: 0.7 ± 0.03 ms WT, 2.6 ± 0.09 ms L858F; at 35°C: 0.2 ± 0.01 ms WT, 1.1 ± 0.09 ms L858F), in agreement with previously published data [7]. At 25°C and 16°C, L858F channels also deactivate significantly more slowly than WT channels at the potentials of -45 and -50 mV.

**Figure 5:**
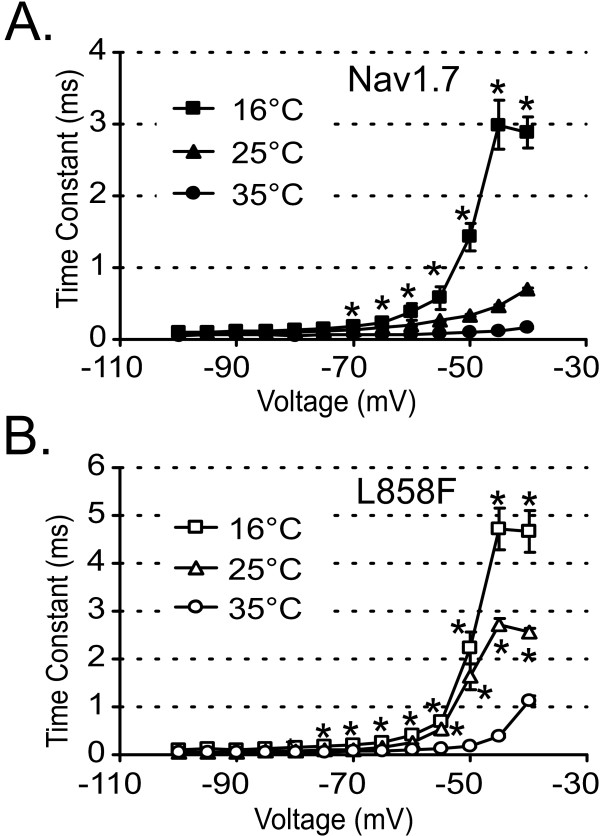
**Cooling increases deactivation time constants for Nav1.7 and L858F**. **A**. Nav1.7 deactivates more slowly at -50 mV, -45 mV and -40 mV when temperatures are lowered from 35°C (filled circles, n = 8) to 25°C (filled triangles, n = 8) and 16°C (filled squares, n = 6). Deactivation time constants were obtained by a single exponential fit of tail currents elicited by repolarization to the indicated potentials from a brief depolarization of 0.5 ms to -20 mV. **B**. L858F deactivates more slowly at potentials ranging from -55 mV to -40 mV when temperatures are lowered from 35°C (open circles, n = 7) to 25°C (open triangles, n = 7) and 16°C (open squares, n = 6). * indicate significant differences to the values at 35°C with p < 0.05, tested with ANOVA and Tukey HSD post hoc analysis.

Figure 5 plots the deactivation time constants for WT and Nav1.7 and L858F channels at 16°C, 25°C and 35°C, and shows that both channels deactivate more slowly with decreasing temperatures. The time constants at 16°C are significantly larger than those at 35°C, for WT at potentials more positive than -70 mV, and for L858F at potentials greater than -80 mV.

## Discussion

Because cooling is known to alleviate symptoms in IEM [4-7,19], a disorder caused by mutations in Nav1.7 [1], we have investigated the effect of temperature on the gating behavior of WT channels and IEM mutant L858F Nav1.7 channels in HEK293 cells stably expressing these channels. Using whole-cell voltage-clamp, we show in this study that lowering the temperature of the recording solution causes a decrease in current density, an increase in ramp currents and a slowing in deactivation for both WT and mutant channels. The V_1/2 _of steady-state activation shows differential temperature sensitivity, and is depolarized at lower temperatures for L858F, but not for WT channels.

A temperature of 35°C was chosen as a reference because it is closest to physiological conditions. The normal temperature of human skin is ~34°C and it can be reduced quickly when exposed to cold water [21]. While cell bodies of DRG neurons are located close to the spinal cord and are therefore at body temperature (~36°C), immunolabelling for Nav1.7 is present along unmyelinated fibers in situ [22], and is predicted to accumulate distally within nerve terminals [11] in the skin, where the channels are exposed to large variations in temperature.

All patients with IEM reported to date experience pain relief by cooling of the limbs. It is not known if all of the mutant Nav1.7 channels respond to cooling in a manner similar to the L858F channels described here. Lowering temperature would be expected to lead to a reduced rate of gating of Nav1.7 channels, as shown previously for neuronal sodium channels in myelinated axons and for muscle sodium channels [23,24]. The decrease in current density that we observed with cooling can be explained at least in part by slowed channel gating. We propose that differential cold-induced modification of gating of mutant Nav1.7 can explain, at least in part, why cooling limbs helps to alleviate the pain.

The hyperpolarizing shift in the V_1/2 _of steady-state activation for L858F compared to WT, at all temperatures tested, is in agreement with our earlier findings [7], and provides a possible explanation for increased excitability in DRG neurons expressing the IEM mutation. However, a reduction in temperature to 16°C causes a significant shift in a depolarizing direction of the V_1/2 _of activation of L858F channels, and this shift is not observed for WT channels. This differential effect causes the activation V_1/2 _for L858F to come closer to the V_1/2 _of WT channels at 16°C. Interestingly, the temperature effect on the slope factor of activation is the same for mutant and WT channels. Increasing the activation threshold of L858F channels is expected to result in a decrease in excitability in DRG neurons expressing the mutant channel, suggesting a possible contribution of this shift of V_1/2 _of activation of the mutant channel to alleviating the symptoms of IEM upon cooling the affected extremities. The V_1/2 _of steady-state fast inactivation, on the other hand, is influenced by temperature in the same way for WT and L858F channels; therefore, a contribution of the shifts in fast inactivation properties to pain alleviation induced by cooling seems unlikely.

Temperature changes have been shown to trigger clinical changes in patients harboring some mutations of the cardiac sodium channel (Nav1.5) and the skeletal muscle sodium channel (Nav1.4) [25-28]. Changes in temperature, for example, evoke symptoms in two inherited diseases of sodium channels, with fever triggering cardiac arrhythmia in Brugada syndrome [27,28] and cold exacerbating muscle weakness or myotonia in paramyotonia congenita [29-31]. The V_1/2 _of activation of WT Nav1.5 does not show temperature sensitivity [32], similar to our findings for WT. The V_1/2 _of activation of WT Nav1.4, however, has been reported to shift in a depolarizing direction upon cooling [33]. One mutation of Nav1.4 causing paramyotonia congenita (I693T [34]) is located in the S4–S5 linker of domain II, only 10 amino acids N-terminal to the amino acid substitution in L858F in Nav1.7 [7], and is located at corresponding position to the IEM mutation Nav1.7/I848T [15]. Patients with the paramyotonia I693T Nav1.4 mutation, and those with the erythromelalgia mutation Nav1.7, both show temperature sensitivity; but while cooling of the IEM patients with Nav1.7/I848T mutations relieves pain, it precipitates symptoms in paramyotonia patients carrying the Nav1.4/I693T mutation. It is interesting in this regard that Plassart-Schiess et al. [33] showed that a reduction in temperature produced a similar effect on WT Nav1.4 and Nav1.4/I693T. Taken together, these data suggest that temperature effects on the behavior of excitable cells may depend on the differential sensitivity of voltage-gated sodium channels and other ionic conductances, which are expressed in these cells.

Nav1.7 responds to slow depolarizations with ramp currents at potentials that are hyperpolarized relative to the threshold of action potential firing, and thus appears to amplify stimuli that, in themselves, do not reach the threshold for the generation of action potentials [15]. L858F has been reported to significantly increase ramp current [7]. We observed this larger ramp current of L858F compared to WT channels at every temperature tested. With cooling, the size of the ramp current increases for both mutant and WT channels. Because the ramp current appears to boost small subthreshold depolarizations [20], we were surprised to see that the increase in ramp current with cooling appeared in mutant as well as in WT channels. As we have noted previously [1] multiple factors, including shift in the voltage-dependence of activation can contribute to hyperexcitability of DRG neurons that express mutant Nav1.7 channels. Thus, we speculate that the depolarizing shift in voltage-dependence of activation of L858F Nav1.7 channels at 16°C, which is predicted to decrease DRG neuron excitability, outweighs the effect of the increased ramp currents.

It should be noted that we have recently shown that the presence of Nav1.8 is critical for rendering mutant Nav1.7 channels expressing DRG neurons hyperexcitable [18]. Nav1.7 appears to be responsible for the initiation of the action potential, whereas the current which underlies the upstroke of action potential is contributed mainly by Nav1.8 [35,36]. The effects of cooling on Nav1.8 are not well understood at this time, but it is possible that altered biophysical properties of Nav1.8, along with altered properties of Nav1.7, contribute to alleviation of pain at decreased temperatures in IEM.

## Conclusion

Temperature shifts have a number of effects on the L858F mutation of Nav1.7, which is known to cause the painful disorder IEM in Chinese and Canadian families. When WT and L858F Nav1.7 channels are compared, the voltage gated sodium current density decreases upon cooling for WT and L858F in the same way. Steady-state fast inactivation shifts in a similar manner for L858F and WT, and ramp currents increase and deactivation slows upon cooling for both L858F and WT. Importantly, steady-state activation of L858F channels is shifted to more depolarized potentials with cooling, whereas the V_1/2 _of activation for WT channels does not change. Thus, lowering the temperature could bring the threshold of activation of the mutant channels closer to that of WT. This effect is likely to contribute to amelioration of pain by cooling of affected extremities in patients with this erythromelalgia mutation.

## Methods

### Plasmids and stable cell lines

HEK293 cells were stably transfected with either Nav1.7_R_, or with the L858F mutation as described previously [7]. Nav1.7_R _is a TTX resistant version of the human Nav1.7 construct that permits recording of Nav1.7_R _currents in isolation from any endogenous TTX-S currents [37,38] (in this study, Nav1.7 (WT) refers to Nav1.7_R_). HEK293 cells were grown under standard culture conditions (5% CO_2_, 37°C) in a 1:1 Dulbecco's modified Eagle's medium and F-12, supplemented with 10% fetal bovine serum and G418.

### Electrophysiology

Whole-cell voltage-clamp recordings [39] of HEK293 cells stably expressing the sodium channels Nav1.7 or the L858F derivatives of Nav1.7 were performed with an EPC-9 and EPC-10 amplifier (HEKA electronics, Lambrecht/Pfalz, Germany) using fire polished 0.8–1.6 MΩ electrodes (World Precision Instruments, Inc, Sarasota, FL, USA). The pipette solution contained (in mM): 140 CsF, 10 NaCl, 1 EGTA, and 10 HEPES; 310 mosmol (pH 7.3, adjusted with CsOH) and the extracellular bath contained (in mM): 140 NaCl, 3 KCl, 10 glucose, 10 HEPES, 1 MgCl_2_, 1 CaCl_2_; 315 mosmol (pH 7.4, adjusted with NaOH for each temperature individually).

The pipette potential was adjusted to zero before seal formation, and the voltages were not corrected for liquid junction potential. Capacity transients were cancelled, and series resistance was compensated by 80–90%. Leakage current was subtracted digitally online using hyperpolarizing potentials applied after the test pulse (P/4 procedure). Currents were acquired using Pulse software (HEKA electronics, Lambrecht/Pfalz, Germany), filtered at 10 Hz and 2.9 kHz in series and sampled at a rate of 20 kHz. For current density measurements, the maximal currents were divided by the cell capacitance, as read from the amplifier. Temperature was controlled using a HCC-100A temperature controller (Dagan, Minneapolis, Minnesota), whereby the bath solution was exchanged with a DHL-A Perfusion system (Shanghai, China).

Voltage protocols were carried out 3 min after establishing cell access. Briefly, standard current-voltage (I-V) families were obtained using 40 ms pulses from a holding potential of -120 mV to a range of potentials (-80 to +40 mV) in 5 mV steps with 5 s between pulses. The peak value at each potential was plotted to form I-V curves. Activation curves were obtained by calculating the conductance G at each voltage V

G=1V−Vrev
 MathType@MTEF@5@5@+=feaafiart1ev1aaatCvAUfKttLearuWrP9MDH5MBPbIqV92AaeXatLxBI9gBaebbnrfifHhDYfgasaacH8akY=wiFfYdH8Gipec8Eeeu0xXdbba9frFj0=OqFfea0dXdd9vqai=hGuQ8kuc9pgc9s8qqaq=dirpe0xb9q8qiLsFr0=vr0=vr0dc8meaabaqaciaacaGaaeqabaqabeGadaaakeaacqWGhbWrcqGH9aqpdaWcaaqaaiabigdaXaqaaiabdAfawjabgkHiTiabdAfawnaaBaaaleaacqWGYbGCcqWGLbqzcqWG2bGDaeqaaaaaaaa@3781@

with V_rev _being the calculated reversal potential. Activation curves were fitted with the following Boltzmann distribution equation:

GNa=GNa,max⁡1+eV1/2−Vmk
 MathType@MTEF@5@5@+=feaafiart1ev1aaatCvAUfKttLearuWrP9MDH5MBPbIqV92AaeXatLxBI9gBaebbnrfifHhDYfgasaacH8akY=wiFfYdH8Gipec8Eeeu0xXdbba9frFj0=OqFfea0dXdd9vqai=hGuQ8kuc9pgc9s8qqaq=dirpe0xb9q8qiLsFr0=vr0=vr0dc8meaabaqaciaacaGaaeqabaqabeGadaaakeaacqWGhbWrdaWgaaWcbaGaemOta4Kaemyyaegabeaakiabg2da9maalaaabaGaem4raC0aaSbaaSqaaiabd6eaojabdggaHjabcYcaSiGbc2gaTjabcggaHjabcIha4bqabaaakeaacqaIXaqmcqGHRaWkcqWGLbqzdaahaaWcbeqaamaalaaabaGaemOvay1aaSbaaWqaaiabigdaXiabc+caViabikdaYaqabaWccqGHsislcqWGwbGvdaWgaaadbaGaemyBa0gabeaaaSqaaiabdUgaRbaaaaaaaaaa@46F5@

where G_Na _is the voltage-dependent sodium conductance, G_Na,max _is the maximal sodium conductance, V_1/2 _is the potential at which activation is half-maximal, V_m _is the membrane potential, and *k *is the slope factor. Inactivation kinetics were assessed by fitting the decay of the current traces with a single exponential fit using PulseFit software (HEKA electronics), revealing the inactivation time constant τ. Ramp currents were elicited by slowly depolarizing voltage ramps, ranging from -120 mV to +20 mV at a rate of 0.23 mV/ms. Ramp current values were expressed as percent of transient peak current obtained during initial I-V families, 3 min after breaking into the cell.

Protocols for assessing steady-state fast inactivation consisted of a series of prepulses ranging from -150 mV to 0 mV lasting 500 ms from the holding potential of -120 mV, followed by a 40 ms depolarization to -20 mV to assess the non-inactivated transient current. The normalized curves were fitted using a Boltzmann distribution equation:

INaINa,max⁡=11+eVm−V1/2k
 MathType@MTEF@5@5@+=feaafiart1ev1aaatCvAUfKttLearuWrP9MDH5MBPbIqV92AaeXatLxBI9gBaebbnrfifHhDYfgasaacH8akY=wiFfYdH8Gipec8Eeeu0xXdbba9frFj0=OqFfea0dXdd9vqai=hGuQ8kuc9pgc9s8qqaq=dirpe0xb9q8qiLsFr0=vr0=vr0dc8meaabaqaciaacaGaaeqabaqabeGadaaakeaadaWcaaqaaiabdMeajnaaBaaaleaacqWGobGtcqWGHbqyaeqaaaGcbaGaemysaK0aaSbaaSqaaiabd6eaojabdggaHjabcYcaSiGbc2gaTjabcggaHjabcIha4bqabaaaaOGaeyypa0ZaaSaaaeaacqaIXaqmaeaacqaIXaqmcqGHRaWkcqWGLbqzdaahaaWcbeqaamaalaaabaGaemOvay1aaSbaaWqaaiabd2gaTbqabaWccqGHsislcqWGwbGvdaWgaaadbaGaeGymaeJaei4la8IaeGOmaidabeaaaSqaaiabdUgaRbaaaaaaaaaa@47FD@

where I_Na,max _is the peak sodium current elicited after the most hyperpolarized prepulse, V_m _is the preconditioning pulse potential, V_1/2 _is the half-maximal sodium current, and *k *is the slope factor.

The rate of deactivation was measured using a short (0.5 ms) depolarizing pulse to -20 mV followed by a 100 ms repolarizing pulse to potentials ranging from -40 mV to -100 mV. Decaying current was then fitted with a single exponential function using PulseFit software (HEKA electronics).

Q_10 _values were calculated as the ratio of the value of parameter X at temperatures T_1 _and T_2_:

Q10=(X2X1)10T2−T1.
 MathType@MTEF@5@5@+=feaafiart1ev1aaatCvAUfKttLearuWrP9MDH5MBPbIqV92AaeXatLxBI9gBaebbnrfifHhDYfgasaacH8akY=wiFfYdH8Gipec8Eeeu0xXdbba9frFj0=OqFfea0dXdd9vqai=hGuQ8kuc9pgc9s8qqaq=dirpe0xb9q8qiLsFr0=vr0=vr0dc8meaabaqaciaacaGaaeqabaqabeGadaaakeaacqWGrbqudaWgaaWcbaGaeGymaeJaeGimaadabeaakiabg2da9maabmaabaWaaSaaaeaacqWGybawdaWgaaWcbaGaeGOmaidabeaaaOqaaiabdIfaynaaBaaaleaacqaIXaqmaeqaaaaaaOGaayjkaiaawMcaamaaCaaaleqabaWaaSaaaeaacqaIXaqmcqaIWaamaeaacqWGubavdaWgaaadbaGaeGOmaidabeaaliabgkHiTiabdsfaunaaBaaameaacqaIXaqmaeqaaaaaaaGccqGGUaGlaaa@3FE9@

Statistical analysis was carried out using SPSS software (SPSS Inc, Chicago, Illinois, USA) performing One-Way ANOVA, and significance at a level of α < 0.05 for multiple comparisons was tested using Tukey HSD post hoc analyses. All data are presented as mean ± SEM.

## Competing interests

The author(s) declare that they have no competing interests.

## Authors' contributions

CH collected, analyzed and interpreted electrophysiological data in China. AL collected, analyzed and interpreted electrophysiological data in the USA. AMR participated in the experimental design of the study and interpretation of the data. SDD-H participated in the experimental design of the study and interpretation of the data. XW conceived the project, participated in the experimental design, supervised the electrophysiological experiments in China. YY conceived the project and participated in the experimental design. SWG conceived the project, participated in the experimental design and interpretation of the data. All authors participated in writing of the manuscript.
